# Treatment of waste stabilization pond effluent using natural zeolite for irrigation potential

**DOI:** 10.1371/journal.pone.0259614

**Published:** 2022-06-03

**Authors:** Kulyash Meiramkulova, Timoth Mkilima, Galym Baituk, Kulzhan Beisembayeva, Abdilda Meirbekov, Anuarbek Kakabayev, Gulmira Adilbektegi, Almas Tleukulov, Gaukhar Tazhkenova

**Affiliations:** 1 Department of Environmental Engineering and Management, Faculty of Natural Sciences, L.N. Gumilyov Eurasian National University, Nur-Sultan, Kazakhstan; 2 Department of Civil Engineering, Faculty of Architecture and Construction, L.N. Gumilyov Eurasian National University, Nur-Sultan, Kazakhstan; 3 Department of Mining, Construction, and Ecology, Sh. Ualikhanov Kokshetau University, Kokshetau, Kazakhstan; 4 Department of Chemistry, Faculty of Natural Sciences, L.N. Gumilyov Eurasian National University, Nur-Sultan, Kazakhstan; 5 Department of Ecology and Chemistry, Khoja Akhmet Yassawi International Kazakh-Turkish University, Turkestan, Kazakhstan; 6 Department of Water Resources and Reclamation, Kazakh National Agrarian University, Almaty, Kazakhstan; Lappeenranta-Lahti University of Technology (LUT University), FINLAND

## Abstract

Direct utilization of treated effluent from natural treatment systems for irrigation can be challenging on sensitive plants due to high levels of salinity. Post-treatment of such an effluent prior to its applicability in irrigation can be of significant importance. In this study, the wastewater from a natural treatment plant was treated using a lab-scale filtration system with zeolite as a filter material. Three different column depths (0.5 m, 0.75 m, and 1 m) were used to investigate the effect of column depth on the treatment efficiency of the media. The suitability of the raw wastewater and the treated effluent from each column for irrigation purposes was investigated. The water quality parameters investigated were; electrical conductivity (EC), total dissolved solids (TDS), sodium (Na+), calcium (Ca2+), and magnesium (Mg2+). From the analysis results, it was observed that the column depth had a significant influence on the removal efficiency of the pollutants. The highest removal efficiency (94.58%) was achieved from the combination of electrical conductivity and 1 m column depth, while the lowest removal efficiency (10.05%) was observed from the combination of calcium and 0.5 m column depth. The raw wastewater fell mostly into a “very high” hazard, which is class four (C4) based on electrical conductivity and class four (S4) based sodium adsorption ratio; making it unsuitable for irrigation purposes. However, when the wastewater was subjected to 1 m column depth, the quality of the treated effluent improved significantly which in turn also improved the suitability of the effluent for irrigation purposes, with percent compliance ranging from 20.19% to 97.54%.

## 1. Introduction

Effluent generated from natural wastewater treatment systems such as waste stabilization ponds is usually saline compared to freshwater due to high concentration levels of dissolved salts. Generally, natural treatment systems are those having little to no dependence on mechanical elements and chemicals to support wastewater purification. The systems mostly depend on plants and microorganisms such as bacteria to decompose and neutralize pollutants in wastewater [1–3].

Unfortunately, the linked high concentrations of salinity in the effluent could pose a significant threat to soil quality and plant growth at large upon its direct application in irrigation without being properly treated [4]. In general, soil salinization is regarded to be among the major threats to plant growth and affects the agricultural sector in many parts of the world [5, 6]. It is also important to note that, natural treatment systems are the most widely used technologies for wastewater treatment in the world [7–9], generating huge volumes of effluents every day. With the fact that fresh water is a vital and scarce resource [10], the reuse of effluents from natural treatment systems becomes an ideal solution for irrigation purposes.

However, studies have observed that crops grown on soils with high electrical conductivity (EC) can significantly reduce their yield [11]. Carbonates, chlorides, sulfates, and bicarbonates of sodium, potassium, magnesium, and calcium are among the salts that can be found in the effluent from waste stabilization ponds. Moreover, when the soil is more saline-sodic, the growth is affected by a combination of high alkalinity, high sodium (Na^+^), as well as high salt concentration [12]. Therefore, it is always necessary to differentiate between soil salinization and soil sodicity. However, salt tolerance among plants differs from one plant to another [13, 14].

Generally, salt tolerance can be defined as the state at which a plant can grow and complete its life cycle on a substrate with high concentrations of levels of soluble salt [15]. Halophytes is the name given to plants that can withstand high concentrations levels of salt in the rhizosphere and grow well [16, 17]. There are many crops with low salinity tolerance including rice (Oryza sativa L.) that has been observed to be highly susceptible to the rhizosphere salinity than other cereals [18]. From rice, it has been observed that high sensitivity mainly occurs at the vegetative and reproductive stages [19].

Among the challenges of salt, accumulation is the tendency of reducing the ability of the plants to uptake water and nutrients, resulting in osmotic or water-deficit stress. For sensitive plants, salt causes injury of the young photosynthetic leaves as well as accelerates their senescence. This is due to the fact that the Na+ cation accumulated in cell cytosol results in affecting the transpiration process of leaves [20]. To determine the suitability of water for use in irrigation, the sodium adsorption ratio (SAR) has been widely applicable as an indicator based on the concentrations of the main alkaline and earth alkaline cations present in the water [21]. Apart from EC and Na^+^, other parameters such as total dissolved solids (TDS), magnesium ion (Mg^2+^), and calcium ion (Ca^2+^) are also important to investigate the suitability of water for irrigation.

Therefore, to make the saline effluent from waste stabilization ponds reusable in irrigation, many treatment technologies have been introduced into the water industry. The technologies include the use of medium filtration and membrane filtration, cation exchange, electrodialysis, sorption, and electrochemical treatments. However, issues related to high capital costs especially due to energy consumption have hindered their application in wider regions [22].

This phenomenon brings significant importance to investigating the potential applicability of low-cost approaches for the treatment of biological treatment plant effluent with respect to irrigation purposes. The application of natural or synthetic zeolites as ion exchangers and adsorbents is regarded as among the relatively economical solutions for treating and reusing wastewater of high salinity [23]. Zeolites as a filter material provide one of the economical technologies and have been widely used in the field of water treatment as ion exchangers and adsorbents [24, 25].

In this study, the potential applicability of zeolites on treating the effluent from a waste stabilization pond for irrigation purposes of low salinity tolerance plants is investigated. Three different column depths (0.5 m, 0.75 m, and 1 m) are used the investigate the influence of column depth on the treatment efficiency of zeolite. Then the effluent from each column is investigated for its potential applicability in irrigation, especially for sensitive plants.

## 2. Materials and methods

### 2.1 Case study description, analytical methods, and wastewater characteristics

The wastewater samples used in this study were collected from the Vingunguti wastewater stabilization ponds in Dar es Salaam, Tanzania, approximately 7.2 km from the city Centre (latitude: 6°50’17.20"S, longitude: 39°14’8.62"E), under the permit given by the Dar es Salaam Water and Sewerage Authority (DAWASA).

Several water quality parameters were investigated in this study, including; EC, Na^+^, TDS, Mg^2+^, and Ca^2+^. The selection of the parameters is based on their significance in determining water suitability for irrigation purposes. In general, Na^+^ was measured using the Sodium-Ion Selective Electrode Method [26], while both Mg^2+^ and Ca^2+^ in water samples were measured using the Ethylenediaminetetraacetic Acid (EDTA) Method [27], with Na_2_EDTA 0.05, Acetylacetone, Tris(hydroxymethyl)-aminomethane (TRIS), Distilled Water and Electrolyte solution L300 as reagents. TDS and EC were determined using the TDS Meter Digital Water Tester (Lxuemlu, Shenzhen, China).

Table 1 presents a summary of the raw wastewater characteristics in terms of minimum (Min), maximum (Max) median, arithmetic mean (AM), and standard deviation (STD) as well as the guidelines for irrigation water as recommended by the Food and Agriculture Organization of the United Nations (FAO) [28]. A maximum EC concentration of 4224 μS/cm was observed from the raw wastewater, with an average concentration of 2478.1 μS/cm.

**Table 1:** Raw wastewater characteristics.

Parameter	Min	Max	Median	AM	STD	Guideline
pH	5.6	8.5	7.2	7.25	0.964	-
EC	1001	4224	2251	2478.10	731.975	1500
TDS	996	2284	1602	1622.20	332.429	1200
Na^+^	115.5	145.7	133.8	131.93	9.226	9
Mg^2+^	8.7	22.5	15.1	14.61	4.095	50
Ca^2+^	6.2	14.3	8.85	9.30	2.344	100

EC in μS/cm, all other parameters in mg/L

### 2.2 Experimental setup

**Fig 1:**
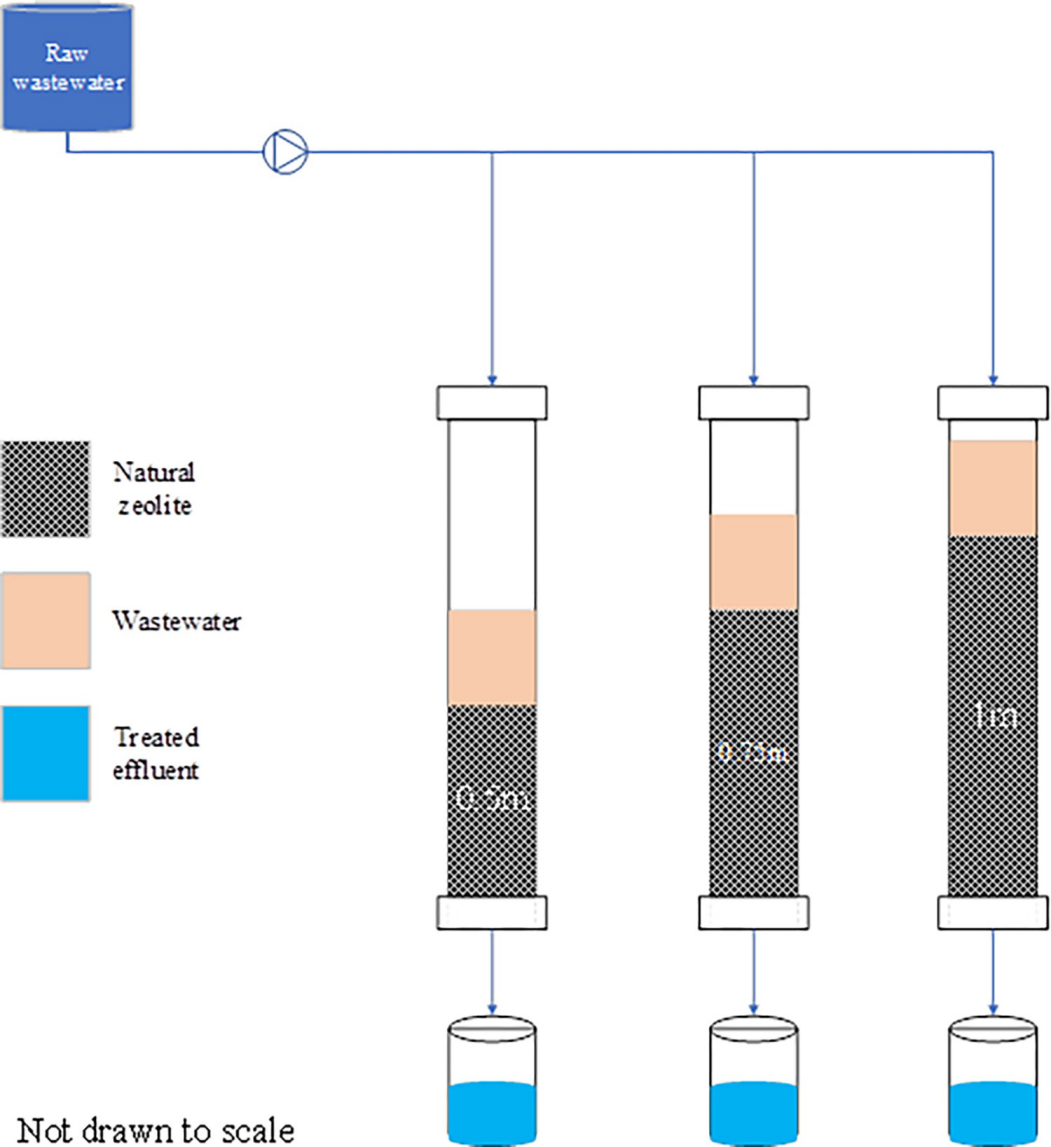
Experimental setup.

Three different fixed-bed columns with 0.5 m, 0.75 m, and 1 m depths were used to investigate the influence of column depth in the treatment efficiency of natural zeolite (Fig 1). The selected column depths serve as both lab-scale and small-scale wastewater treatment systems. The column containers are of Polyvinyl chloride (PVC) material with approximately 5.08 cm in diameter (Table 2). All three columns were packed with natural zeolite adsorbents (clinoptilolite) composed of a microporous arrangement of silica and alumina tetrahedra with an average particle size of 1.5 mm (FM Stock and Supplies, Kenmare, Gauteng, South Africa).

**Table 2:** Physicochemical properties of the zeolite material used.

Parameter	Unit	Value
Particle size	mm	1.5
Bulk density	g/cm^3^	0.74
Particle density	g/cm^3^	1.4
Void ratio	%	48
Surface area	m^2^/g	42
Pore diameter	nm	0.7
Stability in terms of pH	-	Close to neutral
Specific gravity	-	1.89

To allow equal distribution of flow in the columns, the top surfaces of the columns were covered by perforated plates with evenly distributed holes. 100 L storage drum was used to feed the columns at a controlled rate of 0.0035 L/s (based on the amount of wastewater, size of the filter columns, and anticipated daily working hours). To maintain all the solids in suspension, the wastewater was slowly and continuously stirred. The Wet-packing approach of the porous medium was used with the purpose of minimizing layering and air entrapment inside the filing. All three columns were mounted vertically, and glass wool was used at the bottom of the column acting as supporting material of the adsorbent bed. After packing the column, deionized water was passed through the column for some time, followed by the introduction of the feed water. The filtrate samples were collected at a regular time interval. All the experiments were carried out at room temperature (20 to 25 ^0^C).

Table 2 provides a summary of the physicochemical characteristics of the zeolite filter materials used in the study.

### 2.3 Statistical methods

#### 2.3.1 Relationships among parameters

Correlation analysis was among the approaches used to analyze results from the experiments. Correlation matrices were developed to evaluate the strength of the relationship among the studied parameters. A high correlation indicates that two or more variables have a strong relationship with each other. While a weak correlation indicates that the variables are hardly related. Table 3 provides a summary of the interpretation of the correlation indices used in this study.

**Table 3:** Interpretation of the correlation coefficients.

Range of correlation coefficient	Strength of relationship
0–0.29	Weak
0.3–0.49	Moderate
0.5–0.69	Strong
0.7–1	Very strong

#### 2.3.2 Data distribution analysis

Apart from the correlation matrices, box and whisker plots were used to evaluate data distributions among the water quality parameters. The evaluation is based on the distribution of numerical data and skewness through data quartiles (percentiles) and averages. In general, box plots show the five-number summary of a set of data: the minimum score, first (lower) quartile, median, third (upper) quartile, and maximum score. In this study; EC, Na, Mg, Ca, and TDS were analyzed using box and whisker plots.

#### 2.3.3 Adsorption capacity analysis

The adsorption capacity of the filter material (q) was investigated based on the ratio of the amount of adsorbate adsorbed in mg and the amount of adsorbent used for adsorption expressed in gm. Eq 1 provides a summary of the formula used in the computation of the adsorption capacity for the investigated water quality parameters. Some other parameters such as the weight of the material used (M) and the volume (V) of the wastewater subjected to the treatment system were also used.


$$q=\frac{\left[(C_{r}-C_{t})*V\right]}{M}$$ (1)

Where; C_r_ is the average concentration in the raw wastewater and C_t_ is the average concentration in the treated effluent.

#### 2.3.4 Salinity hazard analysis

The salinity hazard zones based on electrical conductivity (EC) were classified into four classes; class one (C1) class two (C2), class three (C3), and class four (C4), ranging from low salinity (C1) to very high salinity (C4). Table 4 provides a summary of the salinity hazard zones based on EC with their interpretations in terms of usability [29].

**Table 4:** Salinity hazard zones: Based on electrical conductivity.

Water class	EC (μS/cm)	Definition
C1—Low salinity	0–250	Water can be used safely
C2—Medium salinity	250–750	Water can be used with moderate leaching
C3—High salinity	750–2250	Water can be used for irrigation purposes with some management practices
C4—Very high	2250–5000	Water cannot be used for irrigation purposes

Table 5 provides a summary of the salinity hazard zones based on Sodium adsorption ratio (SAR) with their interpretations in terms of usability. The salinity hazard zones based on SAR were also classified into four classes and used in the Wilcox diagrams; class one (S1) class two (S2), class three (S3), and class four (S4) ranging from low sodium hazard (S1) to very high sodium hazard (S4) [30].

**Table 5:** Sodium hazard zones: Based on sodium adsorption ratio lines.

Water class	SAR	Definition
S1 low sodium hazard	0–10	Little or no hazard
S2 medium sodium hazard	(10–18)	Appreciable hazard but can be used with appropriate management
S3 High sodium hazard	18–26	Unsatisfactory for most of the crops
S4 Very high sodium hazard	> 26	Unsatisfactory for most of the crops

SAR can be defined as an index used to define the effect of sodium concentration in a sample in relation to calcium and magnesium. More specifically, the SAR index is achieved by diving the square root of 1/2 of the calcium plus magnesium concentrations. Eq 2 provides a summary of the formula used in the computations of SAR [31].


$$SAR=\frac{{Na}^{+}}{\sqrt{\frac{{Ca}^{2+}+{Mg}^{2+}}{2}}}$$ (2)

Furthermore, Wilcox diagrams were plotted from the raw wastewater, effluent from 0.5 m, 0.75 m, and 1 m zeolite columns. The Wilcox plot is a semi-log scatter plot of the "sodium hazard" (SAR) on the Y-axis versus the "salinity hazard" (EC) on the X-axis. It must be noted that the EC is plotted by default on a log scale. The treated effluent suitability for irrigation mainly depends on the concentration of total salinity and sodium related to other ions [32]. Therefore, the diagrams were used to evaluate the risk levels in the raw wastewater and the treated effluent from the three columns.

The irrigation water quality standards were selected to investigate further the quality of the treated effluent. Eq 3 gives a summary of the approach used for the percent compliance calculations.

$$C_{p}(\%)=(\frac{S_{i}-C_{i}}{S_{i}})\times 100$$ (3)

where;

*C*_*p*_, percent compliance,

*S*_*i*_, the recommended standard for an i^th^ parameter,

*C*_*i*_, the concentration of the i^th^ parameter.

## 3. Results and discussion

### 3.1 Water quality characterization

The analysis of the water samples before and after the treatment was successfully executed. In the raw wastewater samples, the average EC concentration was 2478.1 μS/cm, while that of TDS was 1622.20 mg/L. The EC concentration in the raw wastewater falls in class four (C4) based on the salinity hazard zones, with an indication that the effluent from waste stabilization ponds cannot be used directly for irrigation purposes. Average concentrations of 131.9 mg/L, 14.6 mg/L, and 9.3 mg/L were recorded from Na^+^, Mg^2+^, and Ca^2+^, respectively.

**Fig 2:**
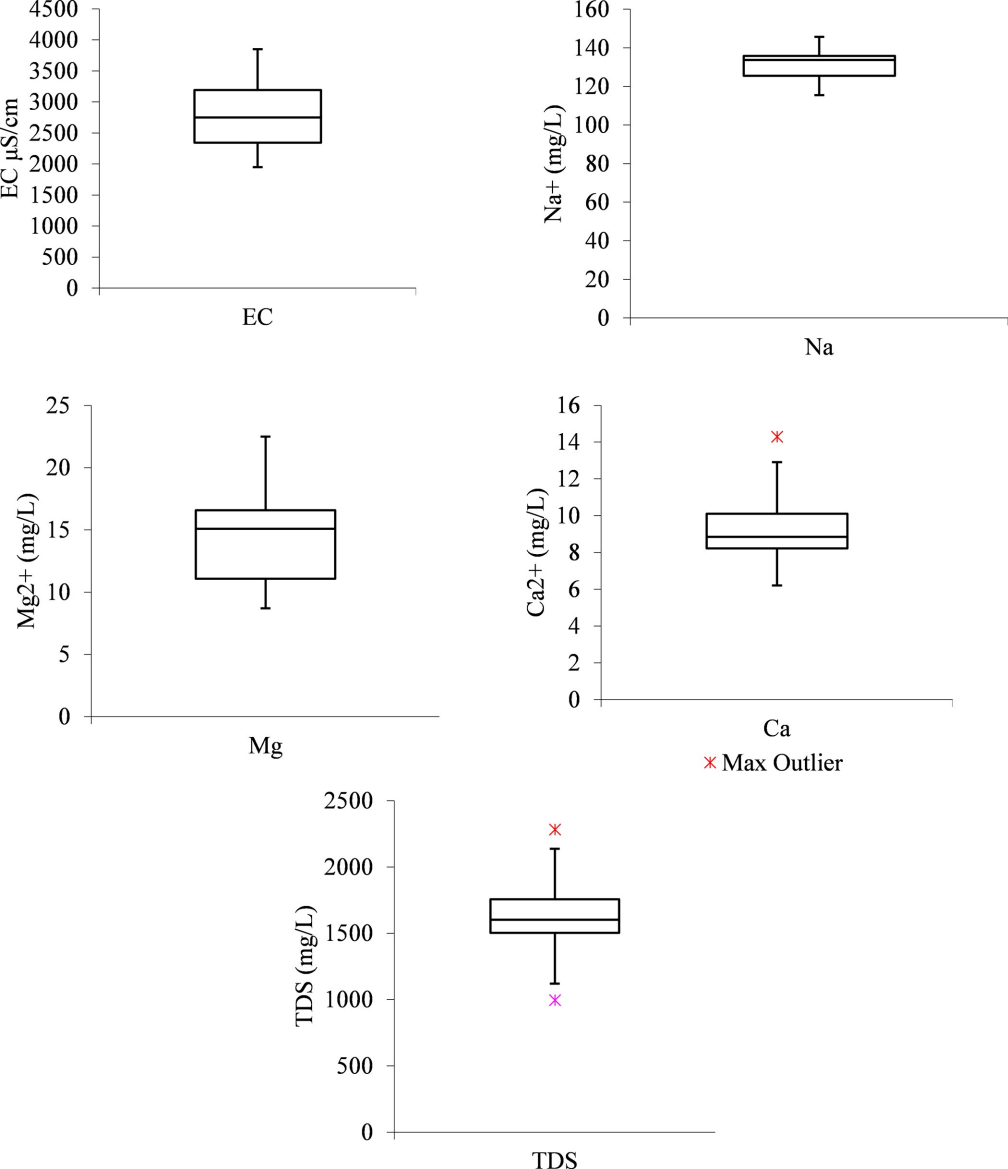
Boxplots from raw wastewater (a) EC (b) Na^+^ (c) Mg^2+^ (d) Ca^2+^ (e) TDS.

From Fig 2, it can be observed that from the EC boxplot, the median line is closer to the middle, indicating that the EC data distribution is symmetric or normal. From the Na^+^ boxplot, it can be observed that the median line closer to the upper quartile with an indication that the distribution of Na^+^ data in the raw wastewater is “negatively skewed”. This means sodium data constituted a higher frequency of low concentration values than the high concentration values.

As observed from the Na^+^ boxplot, a similar case applies to Mg^2+^ concentration data distribution from the raw wastewater. While, from the Ca^2+^ boxplot, the median line is observed to be closer to the lower quartile meaning that the water quality data constitute a higher frequency of more high concentration values than the low concentration values also known as “positive skewness”. Similarly, from the TDS boxplot, the median line is observed to be closer to the lower quartile meaning that the water quality data constitute a higher frequency of high concentration values than the low concentration values (“positively skewed”).

A correlation matrix for the studied water quality parameters in raw wastewater was developed to evaluate the strength of the relationship among them. From Table 6, it can be observed that the general correlation among the parameters ranges from a “strong” to a “very strong” relationship. The highest correlation index of 0.966002 was achieved between Na and EC, followed by 0.945631 between TDS and Na. Also, a very strong correlation can be observed between TDS and EC with a correlation index of 0.944682. The lowest correlation index can be observed between the Mg^2+^ and Na^+^ with a correlation index of 0.598734. However, the index between Mg^2+^ and Na^+^ falls under a strong relationship.

**Table 6:** Correlation matrix from raw wastewater.

	EC	Na^+^	Mg^2+^	Ca^2+^	TDS
EC	1				
Na^+^	0.966002	1			
Mg^2+^	0.650596	0.598734	1		
Ca^2+^	0.837318	0.766448	0.725882	1	
TDS	0.944682	0.945631	0.611418	0.865351	1

In the literature, other studies have also observed a very strong relationship between TDS and EC to the point of recommending the EC to estimate TDS based on the linear relationship as shown in Eq 4 [33, 34]. With the fact that TDS measurement is considered to be a time-consuming process, simplicity is often estimated from EC assuming TDS are predominantly ionic species of low enough concentration to produce a linear TDS-EC relationship [35].


$$TDS\;\left(\frac{mg}{L}\right)=K_{e}\times EC\;(\frac{\mu S}{cm})$$ (4)

Where; K_e_ is a proportionality constant ranging from 0.54 to 1.1.

According to Thirumalini and Joseph [36], that investigated the correlation between EC and TDS in natural waters, it was observed that the correlation index between TDS and EC was 0.63 for samples taken from pollution-free residential areas as well as ranging from 0.59 to 0.93 for samples taken from the textile industrial belt. Therefore, the general strong correlation between TDS and EC observed in the literature agrees with the results obtained from this study.

From Tables 7–9, it can be observed that, when the depth was increased from 0.75 m to 1 m, there was a slight difference in terms of EC and TDS removal in the wastewater. The average EC concentration from the 0.75 m column depth was 487.85 μS/cm, while from the 1 m column depth, the average EC concentration was 378.51 μS/cm. Also, the average TDS concentration from the 0.75 m column depth was 134.36 μS/cm, while that of 1 m column depth was 130.163 μS/cm. The phenomenon suggests that the treatment approach has a gradual removal efficiency as the column depth increases from 0.75 m to 1 m.

**Table 7:** Water quality characteristics from 0.5 m column depth effluent.

Parameter	Min	Max	Median	Mean	STD
EC	901	2403	1202	1352.898	432.395
TDS	980	1282	1101	1107.3	102.363
Na	86.6	127.6	105.65	106.38	11.295
Mg	6.4	10.6	9	8.87	1.416
Ca	4.4	10.3	8.95	8.365	1.918

**Table 8:** Water quality characteristics from 0.75 m column depth effluent.

Parameter	Min	Max	Median	Mean	STD
EC	310	662	501	487.8531	98.452
TDS	88	239.5	123	134.36	42.892
Na	52.9	72.8	63.75	64.07	7.182
Mg	2.06	10.4	6.45	6.51	2.383
Ca	4.5	11.6	7.6	7.81	2.463

**Table 9:** Water quality characteristics from 1 m column depth effluent.

Parameter	Min	Max	Median	Mean	STD
EC	307	442.1	388.5	378.51	44.474
TDS	94	162	137	130.163	28.752
Na	8.5	45.6	22.55	20.75	10.478
Mg	0.9	4.6	2.3	2.5	1.134
Ca	2.5	8.5	5.1	5.15	1.734

**Fig 3:**
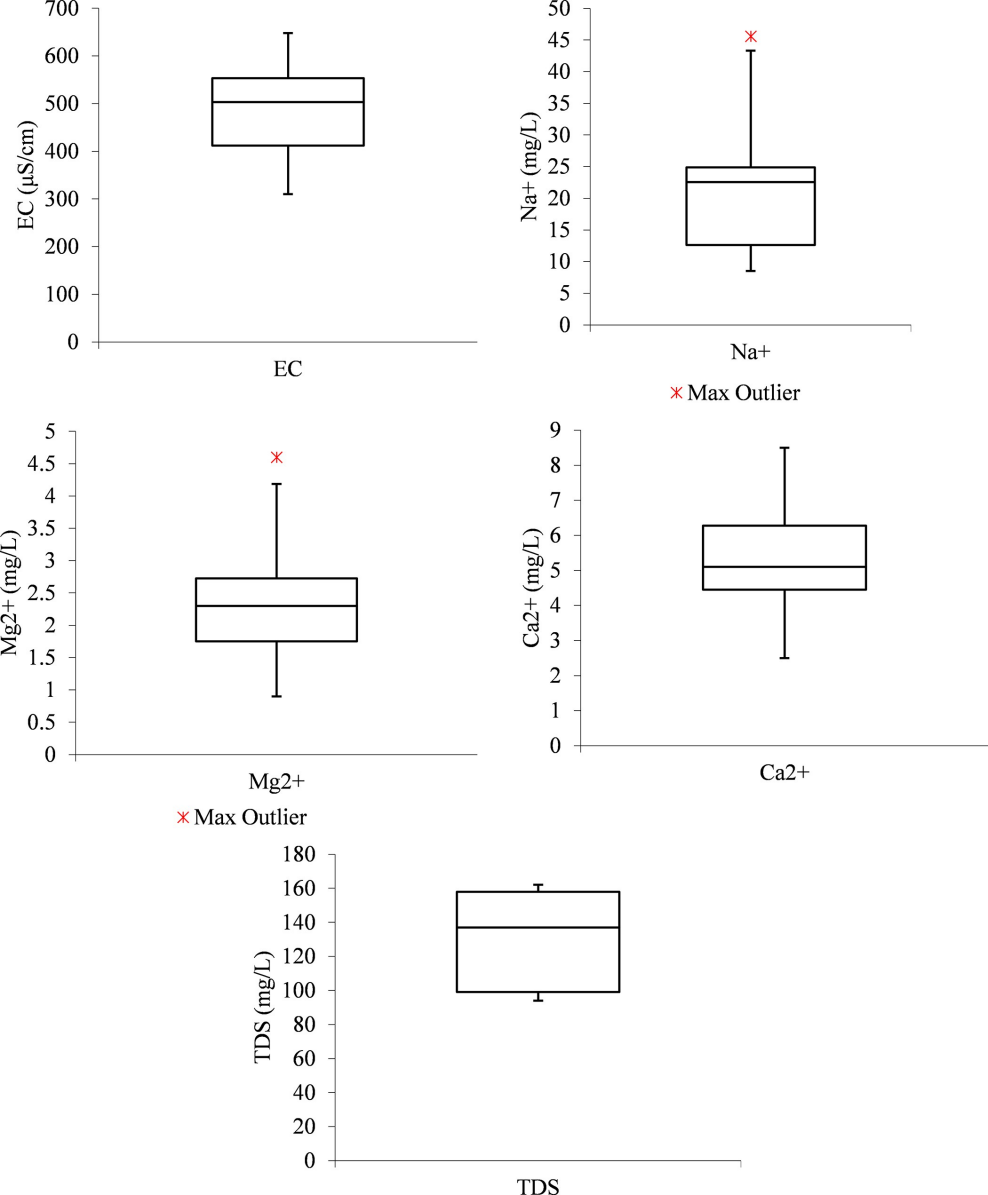
Boxplots from 1 m column effluent (a) EC (b) Na^+^ (c) Mg^2+^ (d) Ca^2+^ (e) TDS.

From Fig 3, the EC, Na^+^ and TDS boxplots, the median line closer to the upper quartile with an indication that the distribution of EC, Na^+,^ and TDS data in the treated effluent using the 1 m column of zeolite is “negatively skewed”. While that of Mg^2+^ is observed to be closer to the middle, indicating that the Mg^2+^ data distribution was symmetric or normal. From the Ca^2+^ boxplot, the median line is seen to be closer to the upper quartile with an indication that the distribution of Ca^2+^ data in the treated effluent using the 1 m column of zeolite is “negatively skewed”. This means the Ca^2+^ data constituted a higher frequency of low concentration values than the high concentration values.

### 3.2 Removal efficiencies

**Fig 4:**
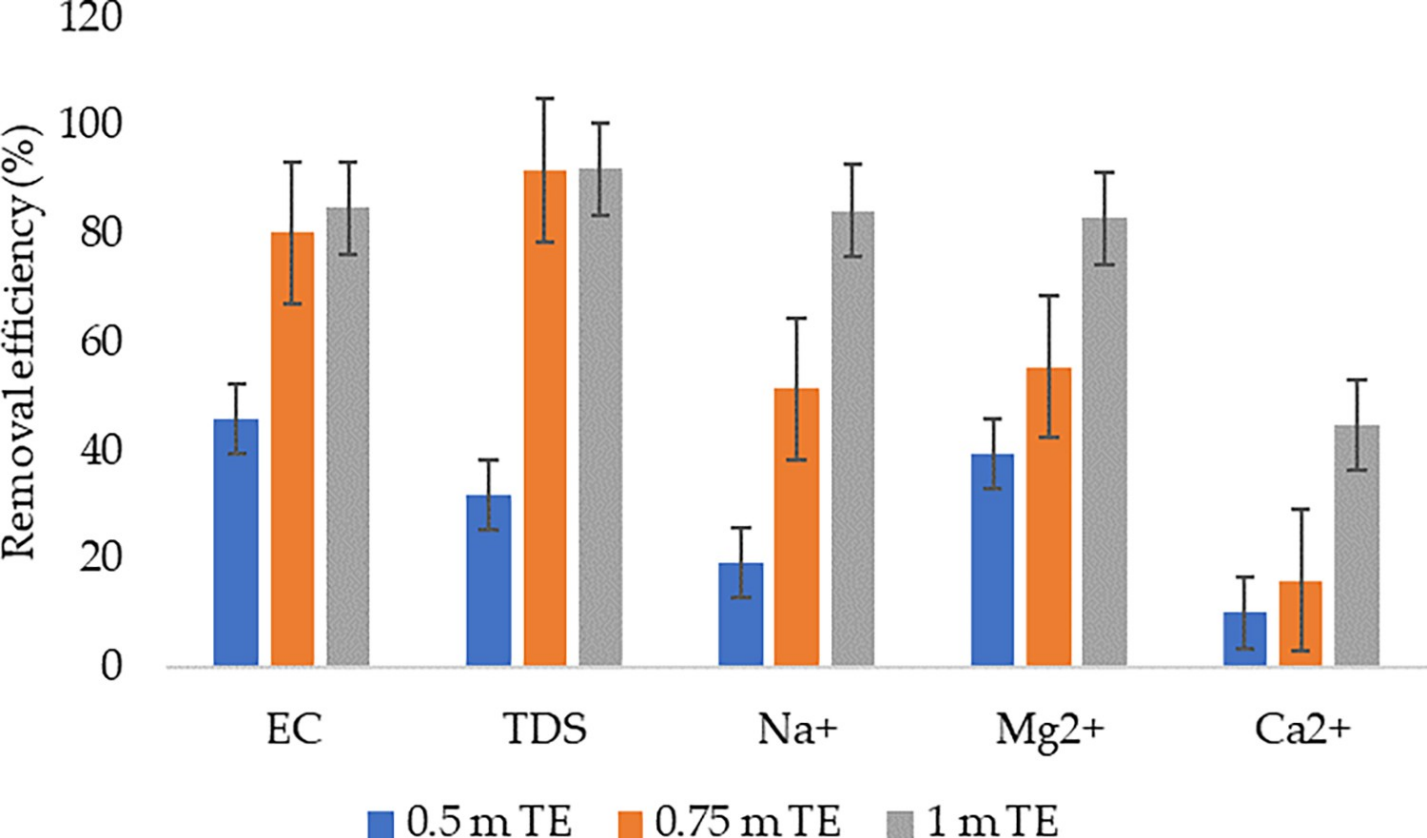
Removal efficiencies from the investigated column depths.

The removal efficiency of the studied parameters was observed to be significantly affected by the column depths; the more the column depth, the higher the removal efficiency (Fig 4). The highest removal efficiency (94.58%) was achieved from the combination of EC and 1 m column depth. This was followed by a removal efficiency of 91.98% from the combination of TDS and 1 m column depth. The lowest removal efficiency can be observed from the combination of Ca^2+^ and 0.5 m column depth. In the literature, natural zeolite has also been observed to be highly efficient in terms of TDS removal. According to [37], which investigated the treatability of brackish groundwater by zeolite filtration in Sumur Tua Wonocolo, Kedewan, Bojonegoro, East Java, a TDS removal efficiency of up to 84% was achieved.

### 3.3 Influence of pH on the general performance of the treatment systems

**Fig 5:**
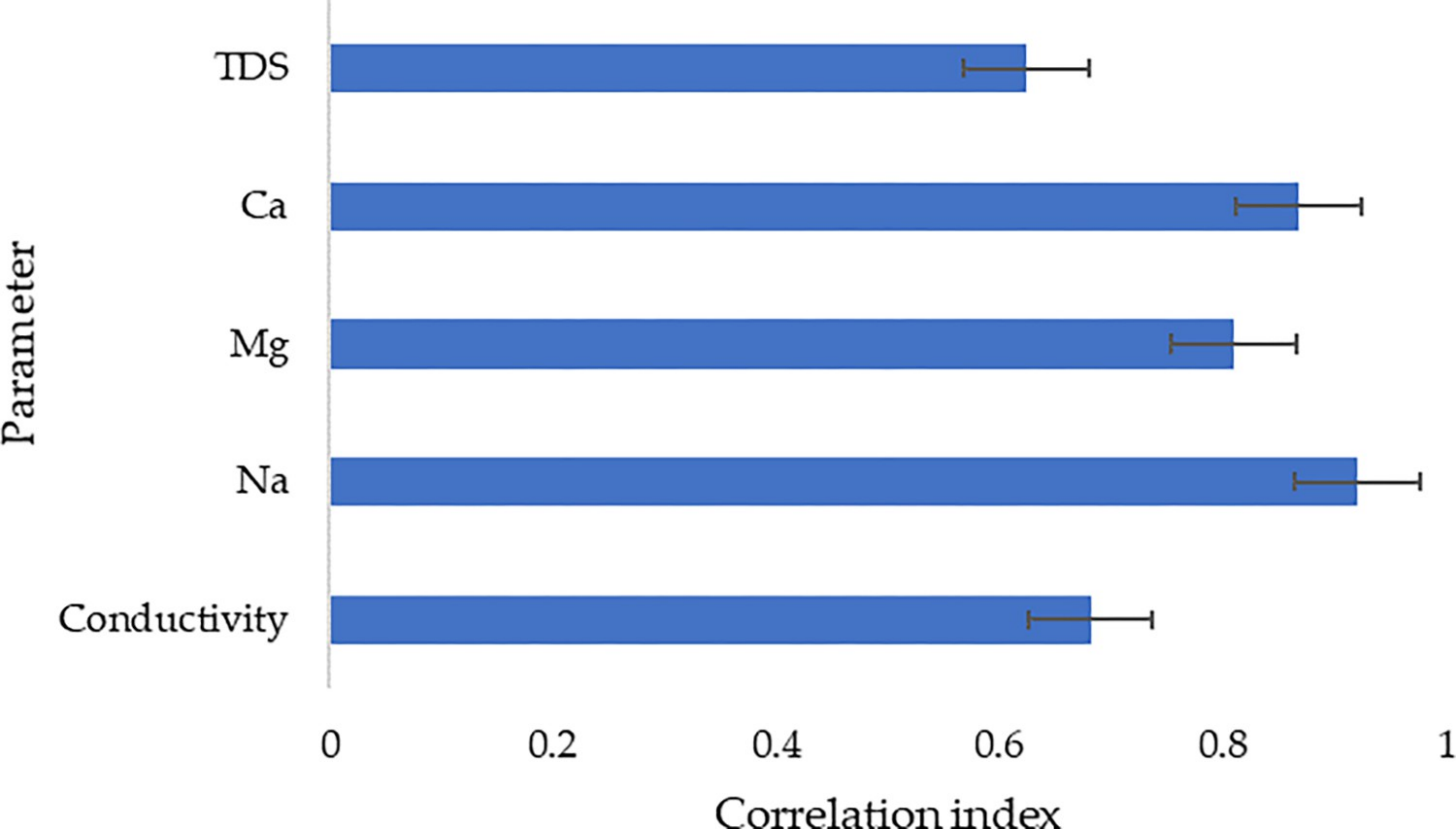
Correlation indices between pH and other water quality parameters.

The influence of pH in the general performance of the filter material for the removal of dissolved solids was investigated based on the correlation indices retrieved from the computed correlation matrices between pH and the water quality parameters. Fig 5 presents the correlation indices in the range of 0 to 1, with 0 being the lowest relationship and 1 being the highest relationship; for total dissolved solids (TDS), calcium (Ca), magnesium (Mg), sodium (Na), and electrical conductivity. The correlation indices ranged from approximately 0.62 to 0.92, which can be termed as a strong to very strong range of correlation. The investigation of the potential influence of pH in the performance of the filter materials in this study is based on the monitored initial pH in raw wastewater. Also, with the fact that pH in the raw wastewater ranged from 5.6 to 8.5, the results were observed when the pH was close to neutral. The phenomenon can be highly linked to the fact pH has a tendency to promote electrostatic interactions between the ions of the pollutant to be adsorbed and the sorbent materials, a phenomenon that makes pH being a crucial parameter in controlling the adsorption processes; as also observed from the study conducted by Ruth Sánchez-Hernández *et al*., [38].

### 3.4 Filter material adsorption capacity based on the investigated water quality parameters

**Fig 6:**
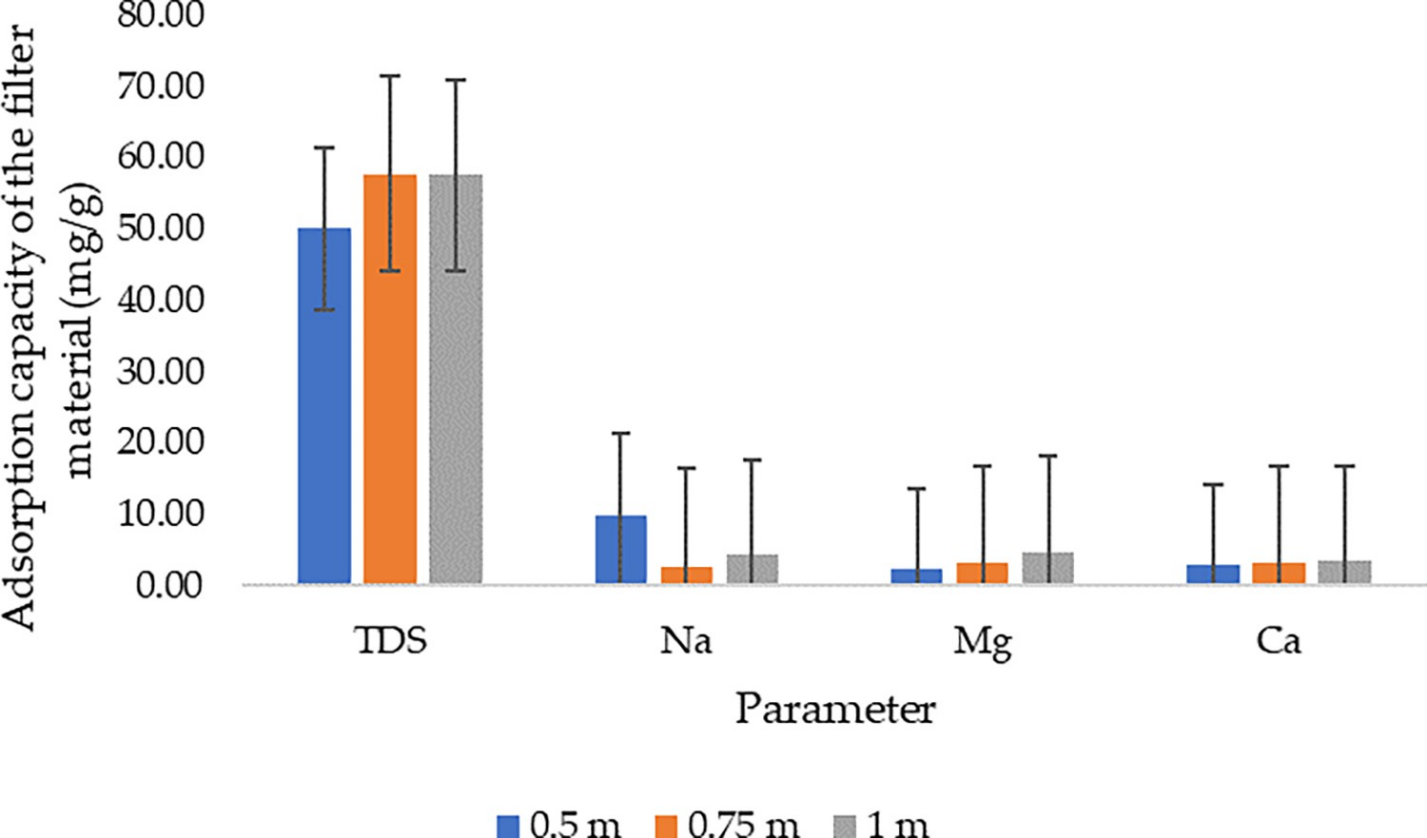
The adsorption capacity of the filter materials.

From Fig 6 it can be observed that there was no specific trend in terms of absorption capacity as influenced by the column depth. Probably, the phenomenon is linked to the fact that the filter material characteristics used in all three columns were similar. For instance, from TDS removal when the wastewater was subjected to the 0.5 m column depth, the average adsorption capacity was 49.96 mg/g, 57.68 mg/g when the wastewater was subjected to the 0.75 m column depth, and 57.55 mg/g when the wastewater was subjected to the 1 m column depth; whereby, on average, 55.06 mg/g adsorption capacity was achieved.

From sodium removal, 9.91 mg/g of adsorption capacity was achieved when the wastewater was subjected to the 0.5 m column depth, 2.63 mg/g when the wastewater was subjected to the 0.75 m column depth, and 4.31 mg/g when the wastewater was subjected to the 1 m column depth. While, from magnesium removal, 2.23 mg/g was achieved from 0.5 m column depth, 3.14mg/g from 0.75 m column depth, and 4.69 mg/g from 1 m column depth. Moreover, from calcium removal, the adsorption capacity of the filter material ranged from 1.63 mg/g to 3.46 mg/g.

**Fig 7:**
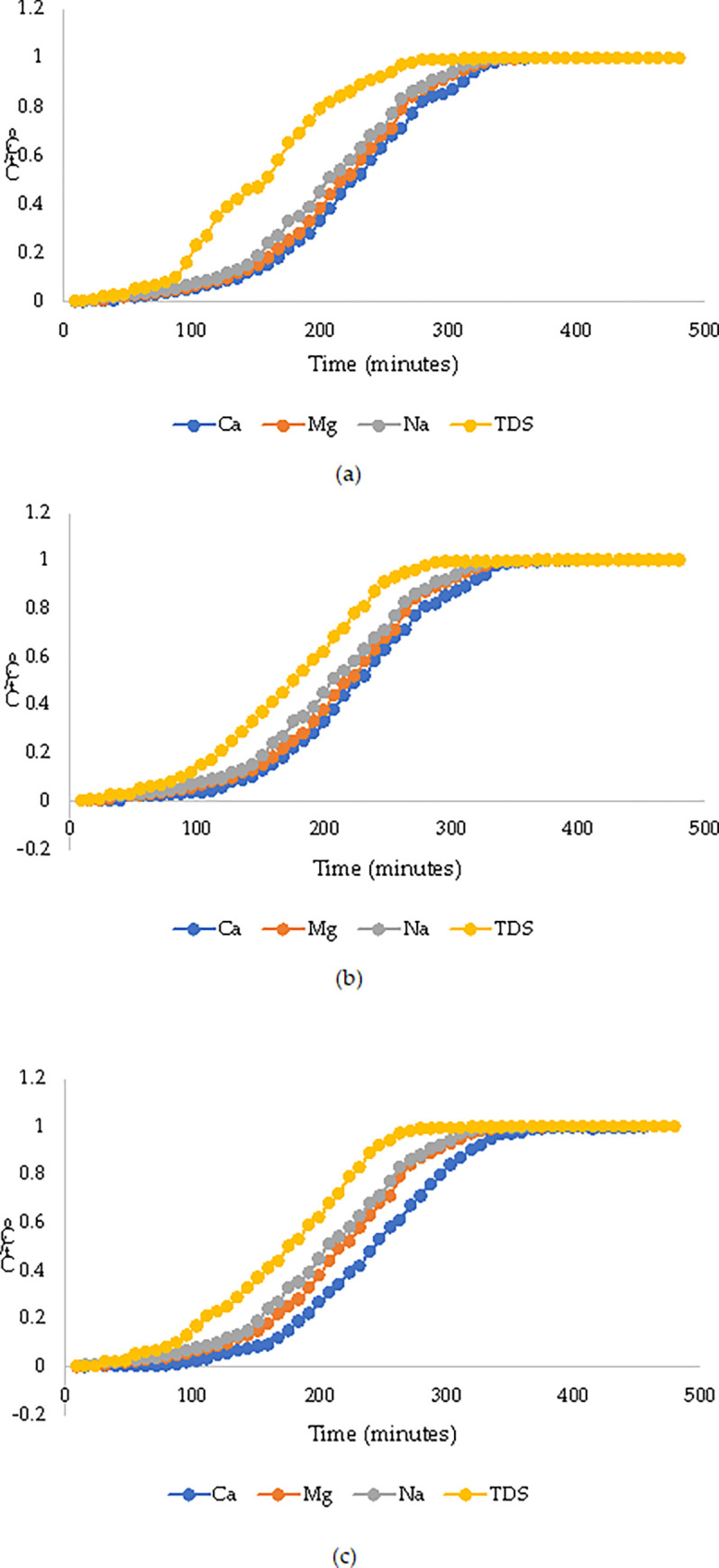
Material breakthrough curves (a) 0.5 m (b) 0.75 m (c) 1 m.

The breakthrough curves for all the investigated column depths and water quality parameters were also plotted based on the ratio of the concentration in the treated effluent at a specific time and the initial concentration in the raw wastewater versus time. From Fig 7, it can be observed that there is a potential relationship between the column depth and the breakthrough times; whereby, when the column depths increase, the breakthrough times also increase. The phenomenon can be likely linked to the fact that the increase in column depth provides space for more filter materials that also take longer to reach the breakthrough time.

### 3.5 Percent compliance

Percent compliance analysis was also executed as part of investigating the quality of the treated effluent with respect to the irrigation water quality standards set by FAO. From Table 10, it can be seen that in the raw wastewater the concentrations of EC, TDS, and Na were above the recommended limits with -65.21%, -35.18%, and 1365.89% compliance, respectively. However, when the wastewater was subjected to 0.5 m column depth, EC and TDS were observed to be complying with the standards having 9.81% and 7.73% compliance, respectively except for Na. Also, when the wastewater was subjected to 0.75 m column depth, the percent of EC and TDS increased to 67.48% and 89.15% respectively, whereby, the percent of Na^+^ increased from -1082% when the wastewater was subjected to 0.5 m column depth to -130.56% from 0.75 m column depth. However, when the wastewater was treated using the 1 m column depth all the investigated water quality parameters were within the recommended limits with percent compliance ranging from 20.19% to 97.54%.

**Table 10:** Percent compliance from irrigation water quality standards.

Parameter	Column depth			
	Raw wastewater (%)	0.5 m (%)	0.75 m (%)	1 m (%)
EC	-65.21	9.81	67.48	97.14
TDS	-35.18	7.73	89.15	96.29
Na	-1365.89	-1082.00	-130.56	20.19
Mg	70.78	82.26	95.00	95.23
Ca	90.70	91.64	94.85	97.54

### 3.6 Irrigation potential analysis

**Fig 8:**
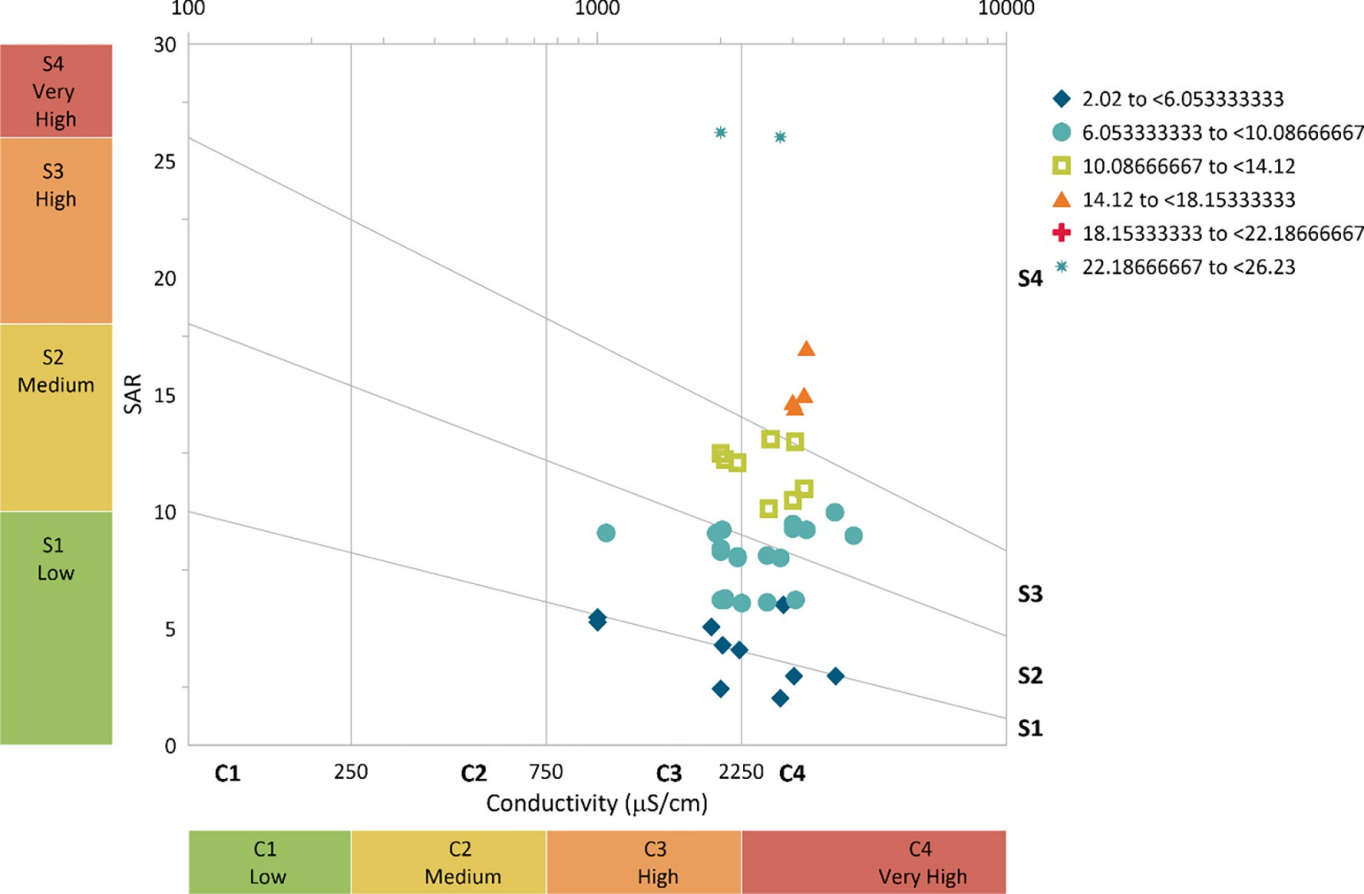
Salinity hazard from raw wastewater.

From Fig 8, it can be observed that the raw wastewater falls under high (C3) to very high (C4) hazards based on the EC. Approximately 68% of the values fall under the very high hazard while 32% fall under the high hazard category. While, based on the SAR, the raw wastewater falls from low (S1) to very high (S4), with most of the values falling under medium (S2) and high (S3). The general phenomenon suggests that raw wastewater is not recommended for irrigation purposes especially for low-salt-tolerance plants based on EC and unsatisfactory for most of the crops based on SAR.

**Fig 9:**
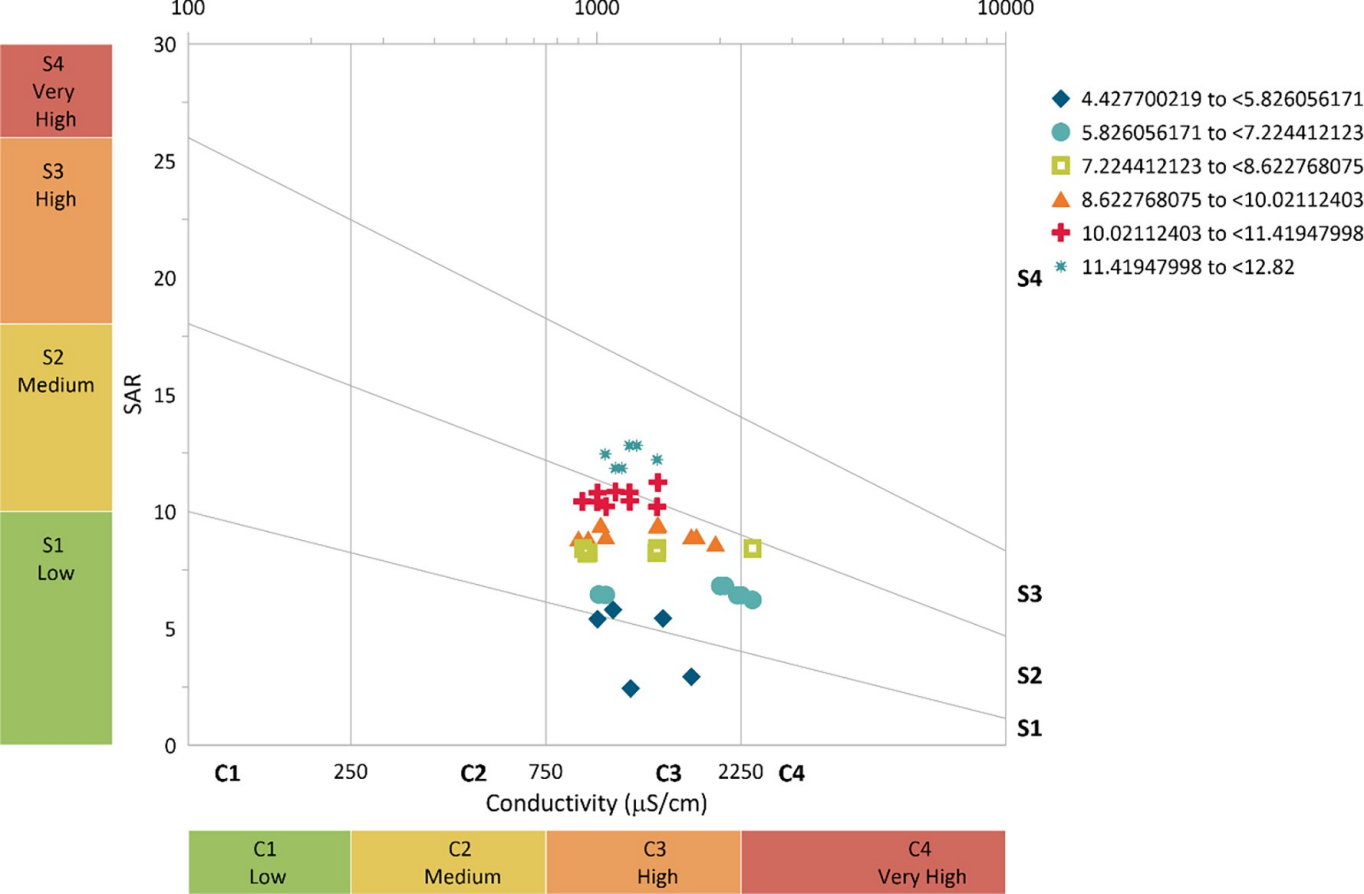
Salinity hazard from 0.5 m column effluent.

From Fig 9, it can be observed that the treated effluent from 0.5 m column of zeolite falls under high (C3) to very high (C4) hazard based on the EC. Approximately 97% of the values fall under the high hazard while 3% fall under the very high hazard category. While, based on the SAR, the treated effluent from 0.5 m zeolite column falls under low (S1) to high (S4), with most of the values falling within medium (S2) hazard. The general phenomenon suggests that the treated effluent from the 0.5 m column is under appreciable hazard but can be used with appropriate management based on SAR and can be used for irrigation purposes with some management practices based on EC.

**Fig 10:**
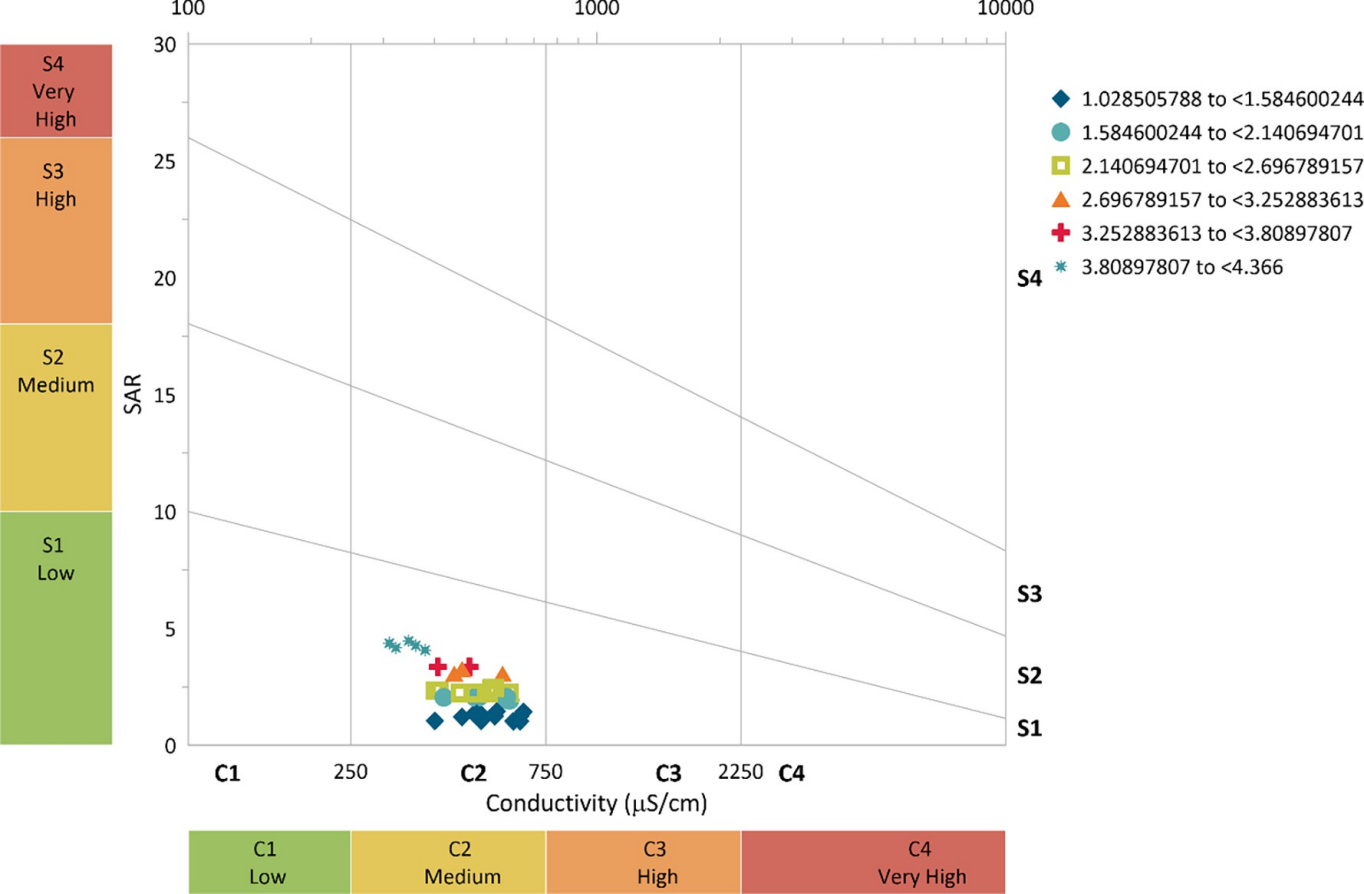
Salinity hazard from 0.75 m column effluent.

From Fig 10, it can be observed that the treated effluent from the 0.75 m zeolite column falls under medium (C2) hazard based on the EC. Almost 100% of the values fall under medium hazard. While, based on the SAR, the treated effluent from 0.75 m falls under low (S1), with very few values getting close to medium (S2). The general phenomenon suggests that the treated effluent from a 0.75 m column can be used for crop irrigation purposes with little or no hazard based on SAR and can be used with moderate leaching based on EC.

**Fig 11:**
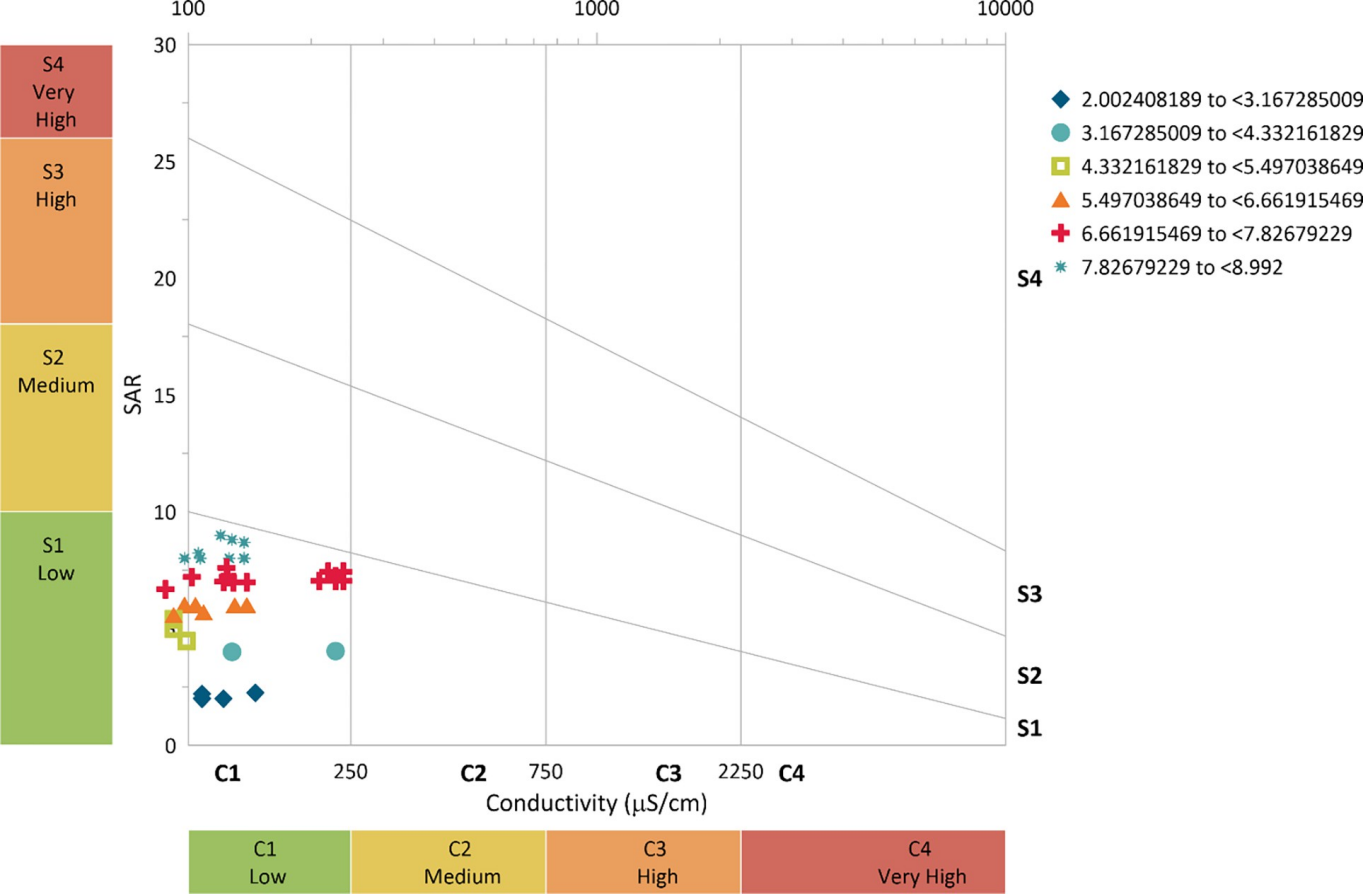
Salinity hazard from 1 m column effluent.

From Fig 11, it can be observed that the treated effluent from the 1 m zeolite column falls under a low (C1) hazard based on the EC. While, based on the SAR, the treated effluent from 1 m column under low (S1), with very little values getting close to medium (S2). The general phenomenon suggests that the treated effluent from the 1 m column can be used for crop irrigation purposes with little or no hazard based on SAR and can be used safely based on EC.

## 4. Conclusions

In this study, the potential applicability of zeolites on treating the effluent from a waste stabilization pond for irrigation purposes has been investigated with three different column depths. A correlation among the studied parameters was observed with the highest correlation index of 0.966002 achieved between Na^+^ and EC, followed by 0.945631 between TDS and Na^+^. Also, the results showed that the pollutants removal efficiency increased with the increase in column depth. Among the studied parameters, the highest removal efficiency (94.58%) was achieved from the combination of EC and 1 m column depth, while the lowest removal efficiency (10.05%) was observed from the combination of Ca^2+^ and 0.5 m column depth. From the hazard analysis, the raw wastewater generally fell into the “very high” hazard class based on both EC and SAR. In that matter, the raw wastewater must be treated further prior to its application for irrigation purposes. However, the status improved after the treatment using different column depths. In that matter, the results from this study revealed further that, it is always important to investigate the quality of effluent from natural treatment systems before subjecting it to any sort of irrigation. Moreover, the use of zeolite can provide one of the efficient approaches to improve the effluent and make it suitable for irrigation.
